# Epileptic Channelopathies and Neuromuscular Disorders in Newborns: A Narrative Review

**DOI:** 10.7759/cureus.43728

**Published:** 2023-08-18

**Authors:** Mohammad N Almohammal

**Affiliations:** 1 Department of Pediatrics, Ministry of Health, Bisha, SAU

**Keywords:** ion channels, newborns, neuromuscular disorders, epilepsy, channelopathies

## Abstract

Neonates can have ion channel abnormalities known as channelopathies, which can impact any organ system. These abnormalities cause seizures, which can result in developmental delays and lead to early death. For a child's long-term neurodevelopment, early identification as a channelopathy is essential to avoid any brain damage. Therefore, this review aims to focus on early diagnostic criteria. Since it might be difficult for doctors to interpret the presenting symptoms of channelopathies, a thorough diagnostic examination that follows a methodical step-by-step procedure is essential. Skeletal muscle fiber and neuron excitability depend on voltage-gated sodium channels. It is now known that mutations in voltage-gated sodium channel genes can cause a growing variety of fatal or debilitating pediatric neurological diseases. Episodic paralysis, myotonia, newborn hypotonia, respiratory impairment, laryngospasm/stridor, congenital myasthenia, and myopathy are examples of muscle phenotypes. There may be a connection between sodium channel malfunction and abrupt infant death, according to recent findings. Numerous epilepsy syndromes and complex encephalopathies are among the manifestations of different channelopathies that are becoming more widely recognized.

## Introduction and background

Neonates can have abnormalities that most typically affect their muscles and brain [1]. Failure of potassium, sodium, chloride, or any other channel can result in channelopathy. It is unknown how frequently channelopathies cause encephalopathy. Genetic flaws, biochemical imbalances, and molecular anomalies can all contribute to neonatal neurological metabolic diseases. Most of these diseases are autosomal recessive or passed down through the mother due to a metabolic pathway enzyme shortage. Newborns' etiology of neurological channelopathy is influenced by specific genes [2,3]. In the current review, Na channelopathy is described in more detail as it is very common. Channelopathies are a group of diseases caused by the dysfunction of ion channel subunits or their interacting proteins.

The greatest diagnostic difficulty and corresponding unmet clinical need for treatment are posed by the recently discovered severe phenotype of skeletal muscle sodium channelopathies present in infancy and childhood [4]. Generic next-generation sequencing panels are being used to detect brain sodium channelopathies, which are primarily severe early-onset epilepsies and epileptic encephalopathies, in young infants [5]. However, adults and older adolescents who might not have received a childhood diagnosis and who might not have had access to the same genetic testing might still receive the incorrect diagnosis and inadequate treatment.

Genetic abnormalities can result in severe neurological conditions, congenital deformities, inborn metabolic problems, and developmental epileptic encephalopathy (DEE) in neonates [6,7]. Other causes of neurological abnormalities in preterm newborns include hyperthyroidism, vitamin B6 insufficiency, and metabolic abnormalities, which are uncommon etiologies. Seizures can appear early in childhood, even right after birth, as a symptom of channelopathies and neurometabolic diseases [8-10]. By recognizing these indicators, an extensive diagnosis can be avoided, triggering the start of therapy and increasing outcomes and long-term results.

Sodium channelopathies of the brain and muscles are uncommon, yet they frequently present to general practitioners. Here, we examine the relationships between clinical manifestations and genetic and pathomechanistic information. Our goals are to increase awareness, aid in diagnosis, encourage efficient care, and maybe lessen morbidity and mortality. This review emphasizes underappreciated juvenile presentations and focuses on the clinical manifestations of SCN4A mutations [11,12].

## Review

Neuromuscular disorders in newborns

Mutations in the *SCN4A* gene lead to skeletal muscle sodium channelopathies, which make it difficult for the muscle to contract or relax. The voltage-gated sodium channel Nav1.4 in skeletal muscle's alpha subunit is encoded by the *SCN4A* gene. Although it is found throughout the sarcolemma, the motor end plate has the highest concentration. The post-synaptic cholinergic receptors are activated by a motor neuron action potential, which encourages acetylcholine release from the nerve terminal. In turn, this prompts Nav1.4 to open, which then causes the propagation of a post-synaptic action potential, which causes muscular contraction. Plasma amino acids, common tandem mass spectrometry, and organic urine acids eliminate these conditions [13,14].

Myotonic presentations

Myotonia, which is characterized by delayed muscular relaxation following a severe contraction, sometimes manifests as painful or stiff muscles or cramps. Muscles in the hands and face are typically more affected than those in the legs by sodium channel-related myotonia. Myotonia alone can be the only symptom of some mutations (sodium channel myotonia), whereas other mutations can cause both myotonia and sporadic muscular weakness (paramyotonia congenita). Myotonic symptoms may be worsened by temperature extremes, especially the cold, as well as by activity or rest after effort. The enlargement of muscles is common [13,14].

Severe neonatal episodic laryngospasm

Babies and young toddlers with SCN4A mutations frequently experience bulbar and respiratory muscle myotonia of varying severity [13]. Severe newborn episodic laryngospasm (SNEL) has been coined to describe the most severe presentations. Infants suddenly develop upper airway muscle myotonia together with respiratory and limb muscle myotonia, commonly leading to stridor (although some occurrences were silent). Life-threatening apnea, hypoxia, cyanosis, bradycardia, and loss of consciousness can occur in different combinations [14,15]. Bulbar dysfunction may cause failure to thrive and necessitate nasogastric and percutaneous endoscopic gastrostomy (PEG) feeding.

Periodic paralysis

In hyperkalemic periodic paralysis (hyperPP), the attacks of flaccid muscular paralysis with high serum potassium levels last minutes to hours and can happen at any time of the day, often beginning in the first decade of life [16]. The mutation that causes this disorder is dominant on SCN4A, with linkage to the sodium channel expressed in muscle. Symptoms are frequently triggered by rest after exertion, low temperatures, or consumption of foods high in potassium. Although myotonia can occasionally happen, paralysis is the most common symptom.

In hypokalemic periodic paralysis (hypoPP), low serum potassium levels cause attacks of flaccid muscular paralysis in patients, which can continue for hours to days and commonly happen at night or right after getting up [16]. As a result, the youngster may struggle to walk in the morning, which may result in absenteeism from school. If symptoms go away by the afternoon, they can be mistaken for school refusal. Resting after exercise and eating meals high in carbohydrates, which stimulate insulin secretion and reduce serum potassium, are triggers. Quadriparesis, which normally spares the face and respiratory muscles, can occur in severe bouts, especially in very young children [17,18].

Congenital myopathy and fetal hypokinesia

Fetal hypokinesia was seen in seven of the affected cases, and they all passed away either during pregnancy or shortly after delivery. The four that made it through suffered delayed motor milestones, neonatal hypotonia, generalized muscle weakness, including facial and neck paralysis, and eventual spinal anomalies. Numerous dysmorphic characteristics, significant infant respiratory insufficiency, and bulbar weakness made PEG feeding and ventilator care necessary.

In the first 10 years, all muscle groups, including the bulbar and respiratory muscles, showed improvement in strength and function. Muscle samples lacked precise diagnostic indicators but were consistent with congenital myopathy. A remarkably similar pattern was corroborated by a second report of three brothers who had compound heterozygous SCN4A mutations [19]. Channel function is lost as a result of SCN4A mutations that cause myopathy; however, there is a clear severity spectrum. Clinical severity and channel malfunction severity have been correlated in in vitro experiments; for example, homozygous total loss of function is fatal.

Congenital myasthenia

The first instance of congenital myasthenia caused by SCN4A was found in a woman who had intermittent respiratory and bulbar paralysis since birth that required ventilator support and resulted in cerebral anoxic damage. Moreover, the other symptoms associated with congenital myasthenia were ptosis, ophthalmoplegia, delayed motor milestones, facial/truncal/limb muscular weakness, respiratory insufficiency, and ophthalmoplegia [19,20]. Despite the fact that acetylcholine is not decreased in this type of myasthenia, pyridostigmine is often ineffective.

Myoclonus dystonia

A movement disease (due to mutation of the *SGCE* gene) called myoclonus dystonia (MD) is characterized by quick, short muscular contractions (myoclonus) and/or persistent twisting and repetitive movements that lead to aberrant postures (dystonia) [21]. The neck, trunk, and upper limbs are frequently affected by the myoclonus jerks that are diagnostic of MD. About 50% of those who are affected experience dystonia in their hands or neck. Dystonia is occasionally the only sign of a movement disorder [22]. There are certain people who tremble. Movement disorders frequently accompany other symptoms. Obsessive-compulsive disorder, depression, anxiety, alcoholism, and panic attacks are examples of non-movement characteristics. MD has no impact on intelligence quotient (IQ), cognition, or lifespan.

Individuals' symptoms might differ greatly from one another, even within the same family. People with MD describe episodic intensification of movement symptoms and variability in symptom severity. MD frequently affects multiple family members and generations, indicating a distinct hereditary component. Even without a familial history, MD can happen. The *SGCE* gene (also known as DYT11) is mutated in about 30-40% of MD sufferers and their relatives. People who have MD may have a novel mutation or have acquired the condition from a parent. Given that MD is dominantly inherited, a child can get the condition by inheriting a mutation from just one of their parents. Father-inherited SGCE mutations almost always result in symptoms in the offspring. Only 5% of children who have the mother's mutation will experience symptoms.

MD can be treated with oral drugs such as benztropine, clonazepam, neuroleptics, dopamine agonists, and possibly gamma-hydroxybutyrate (GHB). The reduction of symptoms after drinking alcohol is a remarkable characteristic in certain MD patients; however, the reaction varies widely, even within particular families. Surgery for deep brain stimulation is starting to look promising. It is possible to investigate complementary therapies, including physical therapy, occupational therapy, and regular relaxation techniques [23,24].

Presentation and treatment of neonatal channelopathy 

Pediatric channelopathies refer to a group of genetic disorders that affect ion channels, which are proteins responsible for controlling the flow of ions (charged particles) across cell membranes. These ion channels play a crucial role in regulating the electrical activity of cells, including nerve and muscle cells. When these channels are dysfunctional due to genetic mutations, they can lead to various health issues, particularly in children.

The classification of pediatric channelopathies is typically based on the specific ion channel affected and the resulting clinical manifestations. Here are some key categories.

Sodium channelopathies:* *These involve mutations in sodium channel genes, affecting the flow of sodium ions across cell membranes. Conditions like congenital long QT syndrome (LQTS), Brugada syndrome, and cardiac conduction diseases fall under this category. These disorders can result in abnormal heart rhythms and sudden cardiac events in children.

Potassium channelopathies: Mutations in potassium channel genes disrupt the regulation of potassium ion flow. This can lead to disorders such as Andersen-Tawil syndrome, causing muscle weakness, developmental delays, and abnormal heart rhythms.

Calcium channelopathies: These channelopathies involve mutations in calcium channel genes, affecting the movement of calcium ions across cell membranes. Examples include Timothy syndrome and congenital myasthenic syndrome, which can lead to neuromuscular and cardiac abnormalities in children.

Chloride channelopathies: Disorders like cystic fibrosis are caused by mutations in chloride channel genes, leading to impaired ion transport across epithelial cells. These conditions primarily affect the respiratory and digestive systems.

Miscellaneous channelopathies: Some conditions do not fit neatly into the above categories but are still caused by ion channel mutations. These include hypoPP and paramyotonia congenita, which involve potassium and sodium channels, respectively, and result in muscle weakness and stiffness.

It is important to note that the clinical presentation of these channelopathies can vary widely, even among individuals with the same genetic mutation. Diagnosis often involves a combination of clinical evaluation, genetic testing, and specialized tests such as electrocardiograms (ECGs) and electromyograms (EMGs). Treatment may include medication, lifestyle modifications, and, in some cases, medical devices or interventions to manage symptoms and prevent complications. Early detection and management are crucial to improving the quality of life and prognosis for children with these conditions.

Table 1 depicts the features of neurological channelopathies in newborns. The inheritance pattern in channelopathy is autosomal dominant, in which the mutations of patients can be de novo or inherited from one of the parents. Neurological channelopathies are rarely associated with other organ systems; an initial magnetic resonance imaging (MRI) at the first seizure reveals unremarkable or mild ventriculomegaly findings but at follow-up, brain atrophy can be seen.

**Table 1:** Presentation and treatment of neonatal channelopathy. AD, autosomal dominant; PHT, phenytoin; OXC, oxcarbazepine; MRI, magnetic resonance imaging; EEG, electroencephalography.

Variables	Channelopathy
Inheritance pattern	AD, de novo
Organ involvement	Brain
Seizure on EEG	Initially fast activity followed up with delta-theta spikes
Seizure type: tonic (general or focal)	Yes
Initial MRI at first seizure	Unremarkable or mild ventriculomegaly
MRI at follow-up	Brain atrophy
Treatment with antiepileptic drugs	Na channel blockers (PHT, OXC)
Outcomes in neurodevelopment	Unremarkable to severe

Seizure Type

Brain MRI typically reveals bilateral hyperintensities in the thalamus and basal ganglia in newborns, which may recover over time [17]. But particular seizure types, like significant myoclonic seizures, and epileptic syndrome may point to a particular metabolic disorder [18]. Neurological disorders, such as infant epilepsy, are frequently caused by metabolic abnormalities. Epilepsy rarely dominates the clinical presentation; instead, other neurological symptoms such as hypotonia and/or vigilance abnormalities are more frequently observed. The alertness of neonates with neonatal channelopathy is similar to that of newborns with neurometabolic abnormalities, according to research [18].

Brain MRI

An MRI scan for neurological channelopathies may typically be normal or exhibit basal ganglia hyperintensity. In a study involving KCNQ2 mutations, the corpus callosum exhibited pronounced corpus callosum thinning and gradual widespread hypomyelination [19,20].

Syndromic Diagnosis

DEEs are age-specific and caused by a variety of etiologies. Growing data point to the importance of genetics in pediatric DEE and other severe neurological illnesses [5,13,21]. Despite the low frequency of each disease, the total incidence is poorly predicted and unknowable. However, in a case series, gene mutations could be extremely uncommon and nonrecurrent [22].

Early infantile epileptic encephalopathy

The neurological condition known as early infantile epileptic encephalopathy (EIEE), which often affects newborns within the first three months of life, is marked by epileptic seizures. EIEE with suppression burst (SB) was first characterized in 1976 [23]. It can either start during infancy or during the first few months of life. According to descriptions of post-anoxic patients, SBs point to a significant cortical network malfunction that may progress into diverse patterns [24]. Infants typically experience tonic seizures, which produce muscle stiffness, but they can also have partial seizures and, less frequently, myoclonic seizures. It is important to identify KCNQ2-related EIEE when there are no obvious genetic inheritance patterns or consanguinity; pathogenic variations may also play a role in the development of epileptic encephalopathies. Encephalopathies are frequently accompanied by anatomical brain malformations or genetic metabolic problems [2].

Early myoclonic encephalopathy

In infants, early myoclonic encephalopathy (EME) happens when a seizure starts before the first two weeks of life. Myoclonus, either prominent or focal, is a common form of seizure. An SB is normally visible on the electroencephalography (EEG); however, it may only happen while you are asleep. Neonatal SB EEG occurs in a pattern of minimal low-amplitude (10 V) discharges that alternate with periods of high-amplitude (>10 V) discharges that are typically slow waves with or without spikes [21,25]. In EEGs, the SB pattern is a distinctive signal. Early epileptic encephalopathy with SB is divided into two separate epileptic syndromes, EIEE and EME, according to the International League Against Epilepsy (ILAE) [23].

EEG, Amplitude-Integrated EEG, and EEG Monitoring

Benign familial newborn seizures are not characterized by any particular EEG attribute, occurring in 50-70% of infants [26-28]. Therefore, the purpose is to identify and confirm the seizure diagnosis in order to give appropriate therapy [29]. Combining continuous EEG (cEEG) with amplitude-integrated EEG (aEEG) enables better seizure identification, including EEG seizures without clinical observation (electrographic seizures) and clinical seizure pattern observation [30]. In a comparable prospective study, Massey et al. observed that of 100 children with acute encephalopathy, 46% exhibited electrographic seizures [31].

There is significant disagreement about and inconsistency with the aEEG background classification [29-31]. Interobserver agreement was low in multicenter research, contrasting the advanced approach with the simple system by al Naqeeb, which was better when using the straightforward method [32]. The outputs of commercial aEEG systems are comparable. Regardless of their level of experience or the incidence of seizures, neonatologists discovered that a straightforward aEEG method provided a better assessment.

Linked channelopathy

The large conductance of calcium and voltage-activated K+ channels' pore-forming component is encoded by KCNMA1. Both excitable and nonexcitable cells in tissues, as well as BK channels, are broadly dispersed. Although a mouse with a worldwide deletion (KCNMA1^−/−^) is viable, numerous organ systems display pathology. Although BK channels play significant roles in animal models, little is known about how they affect humans. The term "KCNMA1-linked channelopathy" refers to a variety of clinically characterized degenerative symptoms caused by 16 uncommon KCNMA1 mutations that were discovered in 37 patients starting in 2005. These mutations include many variants of unknown significance (VUS), as well as gain-of-function (GOF) and loss-of-function (LOF) changes in BK channel activity. Human KCNMA1 mutations are mostly linked to neurological problems like seizures, motor difficulties, intellectual disability, and developmental delays. The variety of symptoms linked to KCNMA1 mutations has grown as a result of the recent identification of additional patients, but brain and muscle dysfunction continues to be the predominant features. New research reveals that the functional big potassium (BK) channel changes caused by the various KCNMA1 alleles may be linked to patient symptoms that are somewhat distinct. However, because the vast majority of KCNMA1 mutations discovered to date have de novo origins and because patients' phenotypes vary widely, it may be possible to find new treatment modalities that have the potential to improve therapeutic efficacy over currently accepted standard regimens by establishing causation between KCNMA1-linked BK channel malfunction and specific patient symptoms [29-32].

Medical treatment

Plans for therapy can include supportive care for muscular abnormalities and depend on the etiology. Oxcarbazepine may be used as the first line of treatment for refractory seizures in KCNQ2 EE [3,4,7,10,21]. Kv7.2 opening Retigabine can reverse the in vitro conductance curve. The advantages and disadvantages in infants still call for a sizable number, nevertheless. Oxcarbazepine and phenytoin sodium channel blockers, which were most successful in 90% of patients in one systemic review examining the efficacy of medications for KCNQ2 seizures, were followed by valproate and phenobarbital in up to 70% of patients. Less than 50% of patients responded favorably to levetiracetam and benzodiazepines [32,33].

Treatment

Pharmacological treatment typically has a major positive impact on myotonic disorders and periodic paralysis. Mexiletine or lamotrigine, often prescribed for myotonic symptoms, is sometimes combined with diuretics and carbonic anhydrase inhibitors to treat periodic paralysis [28, 29]. Treatment can be life-changing for those with severe SNEL. According to some in vitro research, the sodium channel blocker flecainide may be the best treatment option in these circumstances for the prevalent SNEL mutation G1306E [30]. Even though the examination implies a fixed weakness, there is some evidence of the use of similar medications in treating recessive illnesses, thus treatment options should always be taken into account.

Only SNEL has been demonstrated to be a risk factor among muscular sodium channelopathies; however, all of them are debilitating. If treated properly, the prognosis for infants who survive episodic diseases is typically good. School absences are a clear indication that pharmacological treatment is necessary since they can compromise educational ability. The majority of patients continue to be mobile and work full time, but a sizeable proportion develops a fixed proximal myopathy that necessitates walking aids and housing adaptations. There is a discernible severity continuum, as has been shown. The most lethal form of myopathy is congenital, although those who survive it tend to get better within the first ten years and then seem to stabilize [31].

Although the condition has no effect on the heart muscle itself, potassium levels can be severely out of whack in periodic paralysis, particularly in hypoPP, and cardiac arrhythmia related to dyskalemia can be significant. Acute hypoPP episodes may call for oral or IV potassium. This may cause rebound hyperkalemia and iatrogenic death when combined with IV supplementation [33].

Challenges in future

Although sodium channelopathies frequently respond well to pharmaceutical therapy, conducting randomized controlled trials is somewhat complicated by their rarity. This makes it harder to identify best practices and suggests that in the future, trial designs may need to be more creative. The most practical step is frequently to remove unhelpful medications and stratify treatment choices based on genetic etiology, although it is still challenging to establish a regimen that entirely eliminates seizures in DS patients. The creation of novel medicines, with a current emphasis on gene-based methods, presents a significant challenge. Concerningly, the majority of the comorbidities of Down syndrome (DS), behavioral issues, currently have no known treatments at all.

## Conclusions

Significant childhood morbidity and mortality are caused by uncommon but curable skeletal muscle and brain sodium channelopathies. Pharmacological treatments are widely accessible and frequently produce remarkable results when they are tailored to the pathogenic effects of the mutation on channel function. Finding the genetic mutation and understanding how it affects function is critical and has significant therapy implications. The main obstacle to reducing morbidity in muscle sodium channelopathies with young onset may be people's ignorance of how the bodies of infants and young children show clinical symptoms. A thorough early life history of individuals being evaluated as teenagers or adults is crucial when treating brain sodium channelopathies. For a child's long-term neurodevelopment, early identification as a channelopathy and the start of treatment before genetic confirmation are crucial.
